# Tui Na for Chronic Nonspecific Low Back Pain: Protocol for a Systematic Review and Meta-analysis

**DOI:** 10.2196/20615

**Published:** 2021-01-27

**Authors:** Juan Yang, Jeffrey S Brault, Mark A Jensen, Alexander Do, Qingyu Ma, Xuan Zhou, Longbin Shen, Canghuan Zhao, Kwok Chee Philip Cheong, Kejie He, Yu Guo, Zhuoming Chen, Shujie Tang, Yong Tang, Celia Ia Choo Tan, Jiaxu Chen, Brent A. Bauer

**Affiliations:** 1 Division of General Internal Medicine Mayo Clinic Rochester, MN United States; 2 Department of Physical Medicine and Rehabilitation Mayo Clinic Rochester, MN United States; 3 Formula-pattern Research Center School of Traditional Chinese Medicine Jinan University Guangzhou China; 4 Department of Acupuncture The First Affiliated Hospital of Jinan University Guangzhou China; 5 School of Physiotherapy and Exercise Science Singapore General Hospital Singapore Singapore; 6 Department of Orthopedics College of Chinese Medicine Jinan University Guangzhou China; 7 Allied Health SingHealth Group Singapore Singapore

**Keywords:** Tui na, Tuina, low back pain, protocol, systematic review

## Abstract

**Background:**

Chronic nonspecific low back pain (CNLBP) is one of the most common complex pain conditions, and it is strongly associated with high rates of disability. Even though several studies on Tui na for CNLBP have been reported, to our knowledge there has been no systematic review of the currently available publications.

**Objective:**

This study aims to develop a protocol for a systematic review and meta-analysis that will evaluate the effectiveness and safety of Tui na therapy for patients with CNLBP.

**Methods:**

An electronic literature search of PubMed, Embase, MEDLINE, Cochrane Library, Springer, Scopus, World Health Organization International Clinical Trials Registry Platform, Physiotherapy Evidence Database (PEDro), Clarivate Analytics, and Chinese biomedical databases (the China National Knowledge Infrastructure, Wan-fang database, Chinese Scientific Journals Database, and Chinese Biomedical Literature Databases) will be conducted. Studies will be screened by two reviewers independently based on titles and abstracts, followed by a full-text reading with eligibility criteria. Randomized controlled trials involving Tui na for patients with CNLBP will be reviewed. The primary outcomes of the study are improvement of pain, analgesic medication reduction, improvement of functional disability, and degree of satisfaction with the intervention. A secondary outcome is any adverse event of Tui na intervention. Methodological quality and risk of bias will be assessed with the Cochrane Collaboration Risk of Bias Tool. If studies are sufficient, a meta-analysis of the effectiveness will be performed. If possible, we will evaluate publication bias using funnel plots. If substantial heterogeneity between studies is present, and there are sufficient studies, subgroup analyses will be conducted to explain the study findings.

**Results:**

The review database searches will be initiated in December 2020, with findings expected by January 2021.

**Conclusions:**

This protocol will establish a framework of a high-quality literature synthesis on the impact of Tui na treatment in patients with CNLBP. The proposed review will determine whether Tui na is effective and safe for CNLBP patients.

**Trial Registration:**

PROSPERO CRD42020166731; https://www.crd.york.ac.uk/prospero/display_record.php?RecordID=166731

**International Registered Report Identifier (IRRID):**

PRR1-10.2196/20615

## Introduction

Chronic low back pain (CLBP) is one of the most common causes of disability and work absence, affecting 4.2% to 19.6% of individuals, placing a heavy burden on global health care services [1]. About 90% of CLBP patients do not have a clearly identifiable cause of pain, which is classified as chronic nonspecific low back pain (CNLBP) [2,3], a diagnosis based on the exclusion of a certain cause or pathology. CNLBP refers to pain and discomfort located below the costal margin and above the inferior gluteal fold that is not attributed to recognizable or known specific pathology, with (or without) referred leg pain for more than 3 months [4,5]. It is a complex and extremely frequent disorder closely associated with high rates of disability [2]. The symptom onset, recovery, and clinical outcomes of CNLBP are influenced by biological, psychological, and social factors, and therefore its management is complicated and multimodal [6].

Currently, there is not a universally accepted evidence-based treatment approach that has been recommended for patients with CNLBP [7]. Previous clinical practice guidelines focused on relieving pain and pain-related functional disability and relied heavily on pharmacotherapy reduction [6]. Long-term use of analgesics, especially opioids, has been associated with psychological distress like depression and increased risk for other health issues such as falls, fractures, and sexual dysfunction. Thus, current practice is focusing less on pharmacotherapy due to increasing concerns regarding limited efficacy and increased risk. Therefore, nonpharmacological therapies are recommended for CNLBP in current guidelines [8]. This shift in focus has resulted in growing attention on the role that complementary and alternative medicine (CAM) treatments may play [9]. Tui na therapy is one CAM modality that has been widely accepted, especially in Asia [10]. Kong et al [11] reviewed the efficacy of Tui na for LBP and found that it appeared to be an effective therapy. However, to our knowledge, no systematic review and meta-analysis or review protocol relevant to Tui na for CNLBP has been published.

There are many studies on Tui na for patients with CNLBP [11-16]. However, due to nonstandard measurement, nonuniform outcomes, and other factors, these individual studies do not provide sufficient evidence for the impact of Tui na in patients with CNLBP [13,17]. In some countries, Tui na is not even included in the guidelines for the treatment of LBP [18]. Thus, the question of whether Tui na is effective and safe for the management of CNLBP is an important one. A review to help assess the efficacy and safety of Tui na in CNLBP management is therefore important and timely. This paper aims to provide a protocol for a systematic review and meta-analysis on the evidence of Tui na therapy for patients suffering from CNLBP. The primary objective of this review is to identify the effectiveness of Tui na treatment among people with CNLBP. In addition, we will investigate the safety of Tui na in this setting.

## Methods

This protocol was developed to adhere to the PRISMA-*P* 2015 (Preferred Reporting Items for Systematic Review and Meta-Analysis for Protocols 2015) [19]. This study will be conducted in accordance with the PRISMA guidelines [20].

### Study Selection Criteria

The PICOS (patient, intervention, comparison, outcome, and study design) framework will be used to inform the eligibility criteria of studies [21,22]. Studies will be excluded if they meet any of the following criteria: (1) duplicate studies; (2) nonrandomized controlled trials; (3) literature reviews; (4) case reports; (5) studies comparing different types of Tui na; and (6) animal experiments.

### Types of Studies

Any randomized controlled trial (RCT) involving Tui na therapy in the treatment group for patients with CNLBP will be included in this study.

### Participants

Adults (aged 18 years and older) who were diagnosed with chronic, nonspecific (with no diagnosable underlying pathology) LBP will be included in this study. There are no limits on race, gender, age, nationality, etc.

### Intervention

All trials evaluating Tui na intervention will be included. Experimental intervention can be any type of Tui na or Tui na combined with other therapies.

### Comparison

Different control interventions such as no-treatment control, placebo, and other currently used interventions (such as health education, behavior therapy, acupuncture, moxibustion, physical therapies, medications, and gentle touch) will be included.

### Outcomes

Primary outcomes will include improvement of pain, reduction in analgesic medication, improvement of functional disability, and degree of satisfaction with the intervention. A secondary outcome will be any adverse events (AEs) associated with Tui na.

### Search Strategy

An electronic literature search will be applied to the following databases: PubMed, Embase, MEDLINE, Cochrane Library, Springer, Scopus, World Health Organization International Clinical Trials Registry Platform, Physiotherapy Evidence Database (PEDro), Clarivate Analytics, and Chinese biomedical databases (the China National Knowledge Infrastructure, Wan-fang database, Chinese Scientific Journals Database, and Chinese Biomedical Literature Databases), from database inception to December 2020, limited to English and Chinese language only. Peer-reviewed journal articles and reference lists of final included studies, as well as grey literature including conference proceedings, dissertations, and white papers, will be used to help identify all applicable studies for inclusion.

The comprehensive literature search will be designed and carried out by an experienced librarian at Mayo Clinic, Rochester campus, with input from the study’s principal investigator. Controlled vocabulary and keywords will be used to search for publications describing the effect and safety of Tui na on patients with CNLBP.

### Study Selection

**Figure 1:**
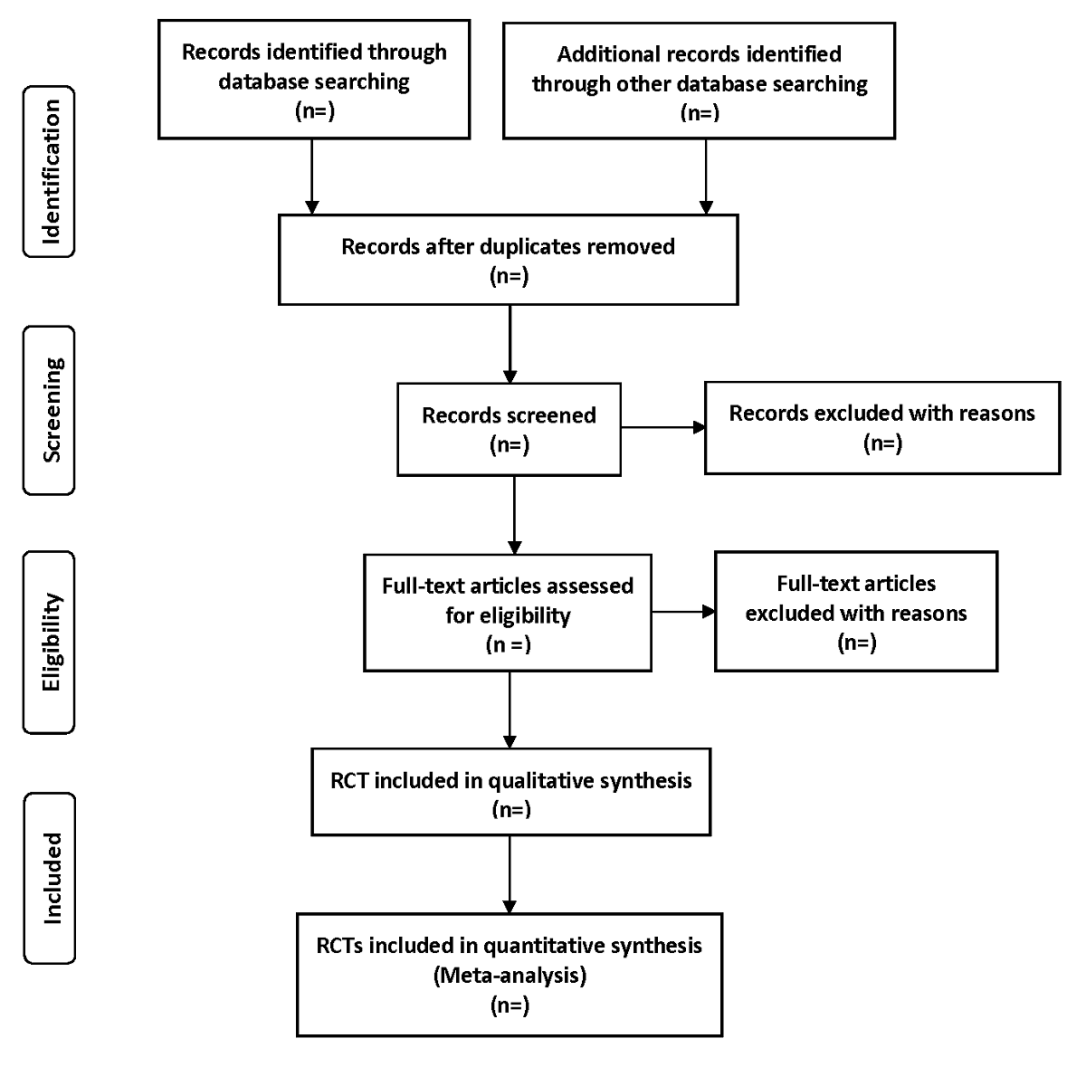
PRISMA diagram of identified studies.

The results of the literature search, including the abstract and citation, will be imported into EndNote X9 (Thomson Reuters Clarivate Analytics), and duplicate studies will be removed prior to the literature screening. Two authors will screen titles and abstracts on inclusion criteria independently. Publications that are not relevant or do not meet the inclusion criteria will be removed. The whole selection process will be presented in the PRISMA flowchart of Figure 1. Any selection divergence will be resolved by the consensus between the two authors or by consulting the original corresponding author.

### Methodological Quality and Risk of Bias Appraisal

To ensure reliability, the risk of bias of each study included in our study will be assessed independently by two authors using the special assessment tool recommended by the Cochrane Collaboration [23], including selection, performance, attrition, detection, reporting, and other biases. An assessment disagreement between the two authors will be discussed between the two authors or by involving a third reviewer until a consensus is reached. Any discrepancies will be resolved through discussion or by involving a third reviewer.

### Data Extraction

Important data associated with our study will be extracted from the included studies using a designed and piloted data collection Excel spreadsheet (Microsoft Corp) by two authors independently. Data will include characteristics of the studies including the first author, published year, sample size, population, and outcome measurement, and detailed information about the interventions such as observation group, control group, provider, frequency, efficacy, AEs, and follow-up.

### Adverse Event Severity

The AE severity is evaluated with the Common Terminology Criteria for Adverse Events (CTCAE) [24]. The CTCAE Scale uses Grades 1 through 5 as follows: Grade 1 (mild AE), Grade 2 (moderate AE), Grade 3 (severe AE), Grade 4 (life-threatening or disabling AE), and grade 5 (death AE). Disagreements will be resolved by discussions. If necessary, the corresponding author will be contacted for clarification.

### Assessment of Reporting Bias

Funnel plots will visually reveal the publication bias if the number of included trials for data analysis is sufficient (a minimum of 10 trials). Egger and Begg tests will be carried out to check the asymmetry of the funnel plot. A symmetric funnel plot represents a low risk of reporting bias, while a dissymmetric funnel plot represents a high risk.

### Data Synthesis

Descriptive analysis or meta-analysis will be conducted according to the interventions, the measurements, and heterogeneity levels. Quantitative findings will be descriptively reported. Continuous variables are analyzed using mean difference and 95% CI, while dichotomous variables will be analyzed using odds ratio. If outcome measure scales are different, the standardized mean difference and 95% CI will be calculated. Study heterogeneity will be calculated within each pairwise comparison by *Q* test and *I*^2^ statistic; higher values indicate higher heterogeneity. *I*^2^=0% indicates no heterogeneity; *I*^2^<25% indicates low, 25%<*I*^2^<50% indicates moderate, while 50%<*I*^2^<75% indicates substantial, and 75%<*I*^2^<100% indicates considerable heterogeneity [25]. If at least 2 included trials are sufficiently homogenous in terms of study design, comparator, and outcome measurement, a meta-analysis will be performed using a random-effects model. Review Manager software (RevMan 5.3) provided by the Cochrane Collaboration will be applied for the meta-analysis. If substantial heterogeneity between studies is present and there are sufficient RCTs, subgroup analyses will be performed to explain clinical heterogeneity effects on study intervention, pain intensity, and measurement tool.

### Ethics and Dissemination

The proposed review will only synthesize previous publications. No research ethics committee review approval is required. Results from this study will be disseminated as a peer-reviewed journal article and presented in conferences.

## Results

This protocol has been registered on PROSPERO (CRD42020166731) on April 28, 2020 [26]. The review database searches will be initiated in October 2020. The literature search strategy is presented in Figure 1. Study results will be submitted for publication in January 2021.

## Discussion

By means of the proposed systematic review, we intend to assess the impact of Tui na in CNLBP. Tui na therapy is a very common, convenient, noninvasive, and relatively inexpensive therapy that has historically been used successfully to treat low back pain [11]. However, Tui na is still not included in the guidelines for the treatment of LBP in most countries, especially those using the mainly Western form of medicine, though other manual therapies are gaining acceptance (eg, chiropractic) [18]. In regard to CNLBP, the evidence is still unclear, preventing its successful adoption into the list of potential treatment options. Thus, the need for this proposed review is clear; to our knowledge, it will be the first to objectively synthesize the currently available publications and evaluate the effect and safety of Tui na for CNLBP patients.

Recognizing that missing data could introduce greater uncertainty and possible bias in estimating the effect of experimental treatment in our meta-analysis, two authors will independently screen, select, and evaluate the data. Any divergence for missing information or unclear information (eg, on study methods or results) will be resolved by consensus between the two authors or by consulting the original corresponding author.

Given the narrowness of our research question, it is possible that only a few RCTs could be included in the proposed systematic review. It is our hope that even should this occur, the research results can still be valuable as a summary of currently available evidence，which may provide some preliminary guidance to inform existing low back pain practice and future research. Finally, to broaden our data and to minimize publication bias, we will conduct the electronic search for studies in both Chinese and English. In the future, as new evidence is made available, the systematic review and meta-analysis is planned to be updated every 1 to 3 years.

This protocol intends to provide a framework of evidence on Tui na therapy for patients with CNLBP that can be used by health care providers worldwide. It is also intended to lay the foundation for future Tui na studies with greater attention focused on remedying deficits found in many of the current studies.

## Abbreviations

AE: adverse event
CAM: complementary and alternative medicine
CLBP: chronic low back pain
CNLBP: chronic nonspecific low back pain
CTCAE: Common Terminology Criteria for Adverse Events
PRISMA-P: Preferred Reporting Items for Systematic Review and Meta-Analysis for Protocols
RCT: randomized controlled trial

## References

[1] Meucci RD, Fassa AG, Faria NMX (2015). Prevalence of chronic low back pain: systematic review. Rev. Saúde Pública.

[2] Hartvigsen J, Hancock MJ, Kongsted A, Louw Q, Ferreira ML, Genevay S, Hoy D, Karppinen J, Pransky G, Sieper J, Smeets RJ, Underwood M, Lancet Low Back Pain Series Working Group (2018). What low back pain is and why we need to pay attention. Lancet.

[3] Chou R, Qaseem A, Snow V, Casey D, Cross JT, Shekelle P, Owens DK, Clinical Efficacy Assessment Subcommittee of the American College of Physicians, American College of Physicians, American Pain Society Low Back Pain Guidelines Panel (2007). Diagnosis and treatment of low back pain: a joint clinical practice guideline from the American College of Physicians and the American Pain Society. Ann Intern Med.

[4] Balagué Federico, Mannion AF, Pellisé Ferran, Cedraschi C (2012). Non-specific low back pain. Lancet.

[5] Airaksinen O, Brox JI, Cedraschi C, Hildebrandt J, Klaber-Moffett J, Kovacs F, Mannion AF, Reis S, Staal JB, Ursin H, Zanoli G (2006). Chapter 4 European guidelines for the management of chronic nonspecific low back pain. Eur Spine J.

[6] Koes BW, van TM, Lin CC, Macedo LG, McAuley J, Maher C (2010). An updated overview of clinical guidelines for the management of non-specific low back pain in primary care. Eur Spine J.

[7] Chenot J, Greitemann B, Kladny B, Petzke F, Pfingsten M, Schorr SG (2017). Non-Specific Low Back Pain. Dtsch Arztebl Int.

[8] Schreijenberg M, Koes BW, Lin CC (2019). Guideline recommendations on the pharmacological management of non-specific low back pain in primary care - is there a need to change?. Expert Rev Clin Pharmacol.

[9] Qi S, Zhang H, Liu X, Zhang G, Guan H, Xie D (2019). Rehabilitation of non-specific low back pain in traditional chinese medicine and conventional medicine. Chinese Manipulation & Rehabilitation Medicine.

[10] Moyer CA, Rounds J, Hannum JW (2004). A meta-analysis of massage therapy research. Psychol Bull.

[11] Kong LJ, Fang M, Zhan HS, Yuan WA, Pu JH, Cheng YW, Chen B (2012). Tuina-focused integrative chinese medical therapies for inpatients with low back pain: a systematic review and meta-analysis. Evid Based Complement Alternat Med.

[12] Wang H (2017). Clinical Study on the Treatment of Nonspecific Low Back Pain with the Method of Relaxing Muscles and Tendons Method Combined with Microwave Therapy. Acta Chinese Medicine.

[13] Zhang Y, Tang S, Chen G, Liu Y (2015). Chinese massage combined with core stability exercises for nonspecific low back pain: a randomized controlled trial. Complement Ther Med.

[14] Zheng Z, Wang J, Gao Q, Hou J, Ma L, Jiang C, Chen G (2012). Therapeutic evaluation of lumbar tender point deep massage for chronic non-specific low back pain. J Tradit Chin Med.

[15] Sun D, Lin D, Cai H, Cai J (2014). Clinical observation of Tuina combined with scale of exercise therapy in the treatment of nonspecific chronic low back pain. The Journal of Neck and Back Pain.

[16] Han H, Chen L, Li L, Fang L, Wang Q (2005). Clinical analysis of manipulation therapy for chronic nonspecific low back pain. Chinese Journal of Convalescent Medicine.

[17] Kong LJ, Fang M, Zhan HS, Yuan WA, Tao JM, Qi GW, Cheng YW (2012). Chinese massage combined with herbal ointment for athletes with nonspecific low back pain: a randomized controlled trial. Evid Based Complement Alternat Med.

[18] National Guideline Centre (UK) (2016). Low Back Pain and Sciatica in Over 16s: Assessment and Management.

[19] Moher D, Shamseer L, Clarke M, Ghersi D, Liberati A, Petticrew M, Shekelle P, Stewart LA, PRISMA-P Group (2015). Preferred reporting items for systematic review and meta-analysis protocols (PRISMA-P) 2015 statement. Syst Rev.

[20] Moher D, Liberati A, Tetzlaff J, Altman DG, PRISMA Group (2010). Preferred reporting items for systematic reviews and meta-analyses: the PRISMA statement. Int J Surg.

[21] Shamseer L, Moher D, Clarke M, Ghersi D, Liberati A, Petticrew M, Shekelle P, Stewart LA, PRISMA- P (2015). Preferred reporting items for systematic review and meta-analysis protocols (PRISMA-P) 2015: elaboration and explanation. BMJ.

[22] Smith V, Devane D, Begley CM, Clarke M (2011). Methodology in conducting a systematic review of systematic reviews of healthcare interventions. BMC Med Res Methodol.

[23] Higgins Julian P T, Altman Douglas G, Gøtzsche Peter C, Jüni Peter, Moher David, Oxman Andrew D, Savovic Jelena, Schulz Kenneth F, Weeks Laura, Sterne Jonathan A C, Cochrane Bias Methods Group, Cochrane Statistical Methods Group (2011). The Cochrane Collaboration's tool for assessing risk of bias in randomised trials. BMJ.

[24] Chen AP, Setser A, Anadkat MJ, Cotliar J, Olsen EA, Garden BC, Lacouture ME (2012). Grading dermatologic adverse events of cancer treatments: the Common Terminology Criteria for Adverse Events Version 4.0. J Am Acad Dermatol.

[25] Ioannidis JPA, Patsopoulos NA, Evangelou E (2007). Uncertainty in heterogeneity estimates in meta-analyses. BMJ.

[26] Yang J, Brault J, Jensen M, Do A, Ma Q, Zhou X (2020). Tuina for Chronic Non-specific Low Back Pain：a systematic review and meta-analysis.

